# Variations of the Organic Matter Composition in the Sea Surface Microlayer: A Comparison between Open Ocean, Coastal, and Upwelling Sites Off the Peruvian Coast

**DOI:** 10.3389/fmicb.2017.02369

**Published:** 2017-12-07

**Authors:** Birthe Zäncker, Astrid Bracher, Rüdiger Röttgers, Anja Engel

**Affiliations:** ^1^RD Marine Biogeochemistry, GEOMAR—Helmholtz Centre for Ocean Research Kiel, Kiel, Germany; ^2^Phytooptics Group, Climate Sciences, Alfred-Wegener-Institute Helmholtz Center for Polar and Marine Research, Bremerhaven, Germany; ^3^Department of Physics and Electrical Engineering, Institute of Environmental Physics, University Bremen, Bremen, Germany; ^4^Remote Sensing, Institute for Coastal Research—Helmholtz Zentrum Geesthacht, Center for Materials and Coastal Research, Geesthacht, Germany

**Keywords:** sea surface microlayer, SML, dissolved organic matter, phytoplankton, peruvian upwelling region, transparent exopolymer particles, air-sea gas exchange, SO243

## Abstract

The sea surface microlayer (SML) is the thin boundary layer between the ocean and the atmosphere, making it important for air-sea exchange processes. However, little is known about what controls organic matter composition in the SML. In particular, there are only few studies available on the differences of the SML of various oceanic systems. Here, we compared the organic matter and neuston species composition in the SML and the underlying water (ULW) at 11 stations with varying distance from the coast in the Peruvian upwelling regime, a system with high emissions of climate relevant trace gases, such as N_2_O and CO_2_. In the open ocean, organic carbon, and amino acids were highly enriched in the SML compared to the ULW. The enrichment decreased at the coastal stations and vanished in the upwelling regime. At the same time, the degradation of organic matter increased from the open ocean to the upwelling stations. This suggests that in the open ocean, upward transport processes or new production of organic matter within the SML are faster than degradation processes. Phytoplankton was generally not enriched in the SML, one group though, the *Trichodesmium*-like TrL (possibly containing *Trichodesmium*), were enriched in the open ocean but not in the upwelling region indicating that they find a favorable habitat in the open ocean SML. Our data show that the SML is a distinct habitat; its composition is more similar among different systems than between SML and ULW of a single station. Generally the enrichment of organic matter is assumed to be reduced when encountering low primary production and high wind speeds. However, our study shows the highest enrichments of organic matter in the open ocean which had the lowest primary production and the highest wind speeds.

## Introduction

The sea surface microlayer (SML) constitutes the uppermost boundary layer between the ocean and the atmosphere. With a thickness varying between tens to hundreds of μm (Liss and Duce, 2005; Cunliffe and Wurl, 2014) the SML is an important factor in global biogeochemical cycles, e.g., influencing gas exchange and the production of sea spray aerosols (SSA) (Cunliffe et al., 2013).

Organic carbon, carbohydrates, and amino acids are often enriched in the SML (Kuznetsova et al., 2004; Reinthaler et al., 2008; Wurl and Holmes, 2008; Engel and Galgani, 2016). Enrichments of gel particles such as the polysaccharidic Transparent Exopolymer Particles (TEP) and the proteinaceous Coomassie Stainable Particles (CSP) have been observed in the SML compared to the underlying water (ULW) in a wide range of ocean systems (Wurl et al., 2011a; Engel and Galgani, 2016). TEP and CSP form abiotically from high molecular weight polymers which are released by phytoplankton and bacteria (Chin et al., 1998; Engel et al., 2004; Verdugo et al., 2004) either through exudation, degradation, or cell lysis (Long and Azam, 1996; Passow, 2002). The accumulation of gel particles and dissolved organic matter (DOM) can result in the formation of a gelatinous biofilm in the SML (Wurl and Holmes, 2008).

Microbial growth is determined by resource availabilities such as those of carbon, nitrogen, and phosphorus, other organic substrates, as well as by the prevailing conditions such as temperature, pH, light, oxygen, and other factors (Madigan et al., 2012). The enrichment of organic matter and gel particles can render the SML a favorable habitat for microbial life. Radiation levels have also shown to be important in the shaping of bacterial communities in the SML of the Southern Baltic Sea (Stolle et al., 2011). In a laboratory study, bacterial amino acid uptake, used as a proxy for bacterial activity, decreased after exposure to intense sunlight (Bailey et al., 1983). Similar photoinhibitory effects are postulated for phytoplankton based on observations of lower phytoplankton concentrations in the SML (Carlson, 1982b).

Mechanisms of microorganisms to defy high irradiation include the production of UV protection proteins and amino acids (Tilstone et al., 2010). The production of mycosporine-like amino acids (MAAs) is used as UV protection in different phytoplankton groups against UV A and B radiation (Lesser, 1996; Bracher and Wiencke, 2000; Zudaire and Roy, 2001). However, some species still experience photoinhibition despite the production of MAAs (Lesser, 1996; Day and Neale, 2002). So far, no consensus has been achieved as to whether the favorable enrichment of DOM and gel particles (Reinthaler et al., 2008; Wurl and Holmes, 2008) or the adverse high radiation (Marumo et al., 1971; Hardy, 1982) are prevailing as microbial community shaping factors in the SML.

Bacterial abundances in the SML are found to be decreased (Engel and Galgani, 2016), to be similar to or slightly increased compared to the ULW (Agogué et al., 2004, 2005a; Obernosterer et al., 2008; Lindroos et al., 2011). Phytoplankton on the other hand is often enriched in the SML (Hardy and Apts, 1984, 1989; Joux et al., 2006) even though some studies also found lower phytoplankton abundance in the SML (Carlson, 1982b) suggesting that the SML is generally a favorable habitat for parts of the microbial community that can withstand the radiation with the help of UV protection mechanisms and can thus benefit from the potentially decreased competition.

The unique properties of the SML regarding its organic matter composition and microbial community as well as its location make it an important, yet not well-characterized, influencing factor in air-sea exchange processes. DOM can influence air-sea gas exchange in two ways—directly by forming a barrier that inhibits the diffusion of gases, and indirectly by changing the surface water properties and thus the gas transfer velocity across the air-sea interface (Davies, 1966; Liss, 1983). DOM has been shown to slow down gas transport rates (Goldman et al., 1988; Frew et al., 1990) and influence the gas exchange even at low concentrations under natural conditions (Schmidt and Schneider, 2011). Thus, the accumulation of DOM in the SML potentially influences the gas exchange between ocean and atmosphere.

Despite the fact that the SML is a distinct habitat, it is clearly linked to the ULW (Kuznetsova and Lee, 2001; Matrai et al., 2008; Lindroos et al., 2011). Through bubble scavenging, turbulence, and other transport processes, organic matter, and organisms from the ULW reach the SML (Bigg and Leck, 2008; Quinn et al., 2014). However, it seems that there are differences in the SML enrichment of organic matter between habitats with different trophic states. Previous studies that investigated the differences in enrichment of TEP, surfactants, and dissolved organic carbon (DOC) for different systems suggested that the enrichment of organic matter in the SML decreases from oligotrophic to more eutrophic environments (Carlson, 1983; Wurl and Holmes, 2008; Wurl et al., 2011b). One of the explanations proposed for this phenomenon was that recycling of DOM is faster in the ULW of eutrophic waters and thus DOM does not have a chance to reach the SML and accumulate (Wurl et al., 2011b).

In this study, we tested whether the concentration and enrichment of organic matter in the SML differ in contrasting oceanic regimes: the open ocean, the coastal waters, and the upwelling region off the Peruvian coast. The SML and ULW were compared with regard to organic matter and microbial community composition. The different regions were chosen according to chlorophyll a (chl *a*) concentration and their proximity to the coast and to the upwelling waters off the coast. We took SML and ULW samples in the Peruvian upwelling regime in October 2015 and analyzed the organic carbon and nitrogen content, carbohydrates, amino acids, gel particles, overall phytoplankton biomass as well as bacterial and phytoplankton (nano- and picophytoplankton) abundances. This wide range of parameters allows a profound analysis of the composition and degradation state of the organic matter across the different water masses and its fate at the boundary layer between the ocean and atmosphere.

## Materials and methods

### Sampling site and sampling

**Figure 1:**
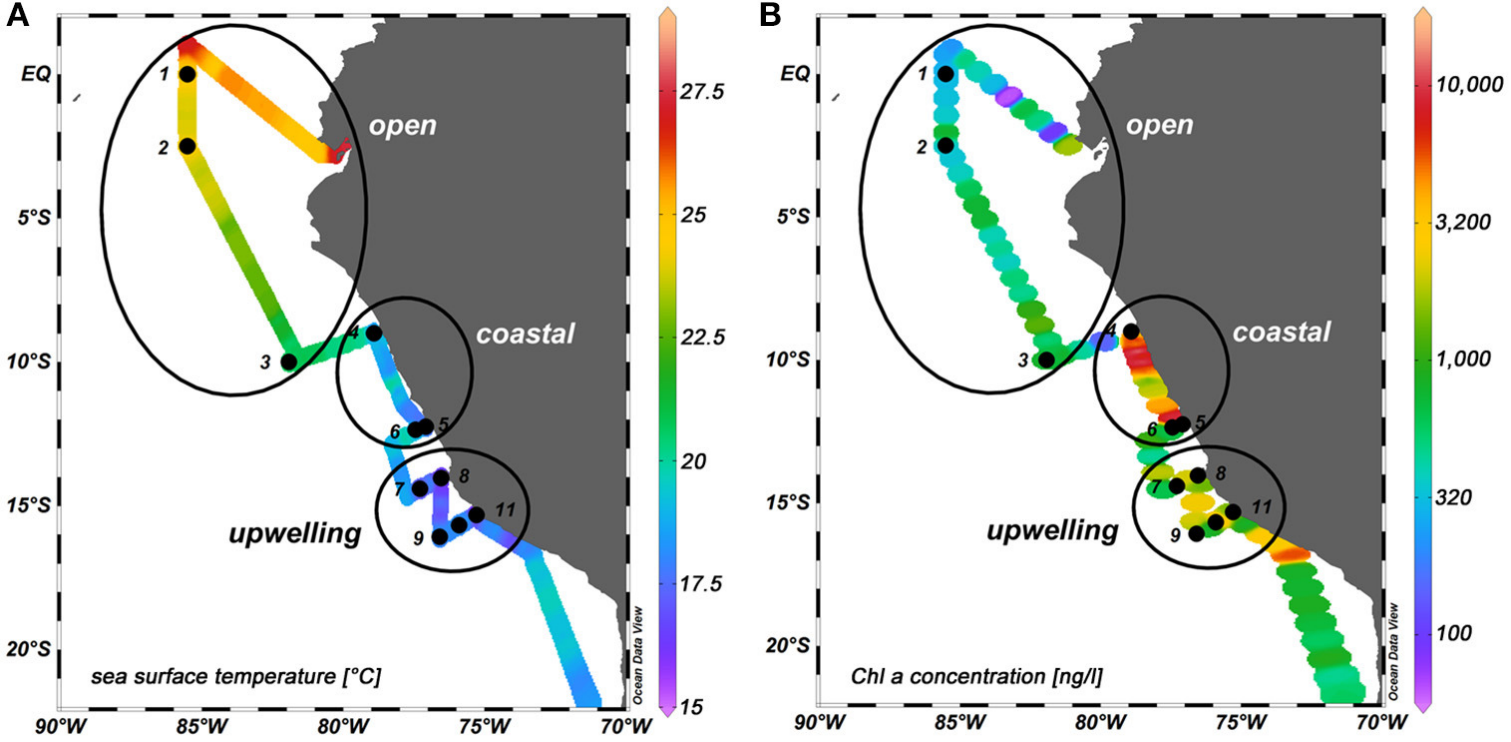
SO243 cruise track. Black dots: sampling sites. The numbers indicate the station numbers. Black circles: regimes that were distinguished according to their location, phytoplankton biomass (in terms of surface chl *a* concentration) and anthropogenic input of nutrients (see section The SML as a Distinct and Variable Habitat and Its Impact for details). **(A)** Water temperatures at ~2 m depth in °C. **(B)** Chl *a* concentration in ng l^−1^ at 6 m depth.

Samples were collected during the SO243 cruise on the RV *Sonne* to the Peruvian upwelling region from 4 to 22 October 2015. 11 stations were sampled between 0°S and 15.7°S and 85.5°W and 75.3°W (Figure 1) from the surface microlayer (SML) and the underlying water (ULW; typically at about 20 cm).

The SML was sampled from a rubber boat using a glass plate sampler (Harvey, 1966). The glass plate is made of silicate glass with the dimensions 50 × 26 cm which lead to an effective sampling surface area of 2,600 cm^2^ considering both sides. Sampling was conducted when the rubber boat was located 0.5 nautical miles away from the research vessel, slowly moving into the direction of the wind to avoid contamination. The SML samples were taken by immersing the glass plate perpendicular to the sea surface and withdrawing it at a controlled rate of ~17 cm s^−1^. The sample was then collected from the glass plate using a Teflon wiper into an acid cleaned, rinsed glass bottle.

All sampling material was cleaned with acid (10% HCl) and purified water (MilliQ) before usage. To avoid contamination during the transport, the glass plate and the Teflon wiper were copiously rinsed with seawater at the sampling site. The first 10 ml of the sample were used to wash the glass bottle in which the sample was collected.

The thickness of the SML was calculated with the following formula (Carlson, 1982a; Cunliffe and Wurl, 2014):

(1) $$\begin{array}{l} \text{d }=\text{ V}/\left(\text{A x n}\right), \end{array}$$

where *V* is the total SML volume collected, *A* is the sampling area of the glass plate considering both sides and *n* is the total number of dips that were used to collect water from the SML at the respective station. About 1.2 l of water from the SML was collected at each station. The same amount was also collected from the ULW. The water from the ULW was collected in two acid cleaned (10% HCl) and MilliQ rinsed plastic bottles attached to a LIMNOS water sampler (Hydro-Bios, Germany).

### Total organic carbon (TOC) and dissolved organic carbon (DOC)

Samples for TOC and DOC were collected in combusted glass ampoules (8 h, 500°C). Samples for DOC were filtered through polyethersulfone filters with a pore size of 0.45 μm and a diameter of 25 mm (Macherey-Nagel).

All samples were acidified with 20 μl of 30% HCl (Suprapur®, Merck), flame sealed, and stored at 4°C in the dark. The samples were analyzed using the high-temperature catalytic oxidation method in a TOC analyzer (TOC-VCSH, Shimadzu) (Sugimura and Suzuki, 1988) with modifications. Calibration of the instrument was conducted every 8–10 days by measuring standard solutions of 0, 500, 1,000, 1,500, 2,500, and 5,000 μg C l^−1^ prepared from a potassium hydrogen phthalate standard (product number 109017, Merck). On each measurement day, the instrument baseline was set with purified water (MilliQ). Deep-sea water with a known TOC/DOC concentration (Dennis Hansell, RSMAS, University of Miami) was used to verify results. Additionally, two internal standards prepared from the same potassium hydrogen phthalate solution were used each measurement day. Their concentrations were within the range of the sample concentrations. The internal standards were used in the beginning and at the end of the measurement day to detect drift within the measurements. The samples were diluted with two parts of purified water (MilliQ). One percent of 1 M HCl was added to acidify the samples. Generally five injections were used, if the four best samples showed a variance >1% however, additional samples (up to eight) were measured.

### Total nitrogen (TN) and total dissolved nitrogen (TDN)

TN and TDN were measured at the same time as TOC and DOC, respectively, with a TNM-1 detector on the Shimadzu analyzer. The instrument was calibrated every 8–10 days by measuring standard solutions of 0, 100, 250, 500, and 800 μg N l^−1^, prepared from potassium nitrate (Suprapur®, Merck product number 105065).

### Total and dissolved combined carbohydrates

Samples (20 ml) for total and dissolved hydrolysable carbohydrates >1 kDa (TCHO and DCHO, respectively) were filled into combusted glass vials (8 h, 500°C). The samples were stored at −20°C until analysis. Prior to filling the glass vials, samples for DCHO were filtered through 0.45 μm Acrodisc® syringe filters (Millipore). For the analysis, high performance anion exchange chromatography with pulsed amperometric detection (HPAEC-PAD) was applied on a Dionex ICS 3000 ion chromatography system (Engel and Händel, 2011). Samples were desalinated using membrane dialysis (1 kDa MWCO, Spectra Por) at 1°C for 5 h. Subsequently, the samples were hydrolyzed for 20 h at 100°C with 0.8 M HCl final concentration and neutralized with acid evaporation (N_2_, for 5 h at 50°C). Two replicates per sample were analyzed.

### Total and dissolved amino acids

Total hydrolysable amino acids (TAA) were determined from 5 ml of sample which were filled into precombusted glass vials (8 h, 500°C) and stored at −20°C until analysis. Samples for dissolved hydrolysable (DAA) and free amino acids (FAA) were filtered through 0.45 μm Acrodisc® syringe filters (Millipore) and then stored at −20°C until analysis. The analysis was modified from established methods (Lindroth and Mopper, 1979; Dittmar et al., 2009). Samples were measured in duplicates. The samples were hydrolyzed at 100°C for 20 h with HCl (Suprapur®, Merck) and neutralized by acid evaporation under vacuum at 60°C in a microwave. Remaining acid was removed with water.

Samples were analyzed by high performance liquid chromatography (HPLC) using an Agilent 1260 HPLC system. Prior to the separation of 13 amino acids with a C^18^ column (Phenomenex Kinetex, 2.6 μm, 150 × 4.6 mm), in-line derivatization with o-phthaldialdehyde and mercaptoethanol was performed. Analysis was done using a gradient with solvent A containing 5% acetonitrile (LiChrosolv, Merck, HPLC gradient grade) in sodiumdihydrogenphosphate (Suprapur®, Merck) buffer (pH 7.0) and solvent B being acetonitrile. A gradient from 100% solvent A to 78% solvent A was produced in 50 min. FAA were measured from DAA samples without prior hydrolysis in separate analyses.

### Gel particles

The abundance and area of gel particles was measured microscopically (Engel, 2009). Depending on the concentration of gel particles in the water, 10–30 ml of the sample were filtered onto a 0.4 μm Nucleopore membrane (Whatman). TEP were stained by applying 1 ml of an Alcian Blue solution to each filter for 3 s. CSP were stained accordingly with 1 ml Coomassie Brilliant Blue G (CBBG) applied for 30 s. Filters for both types of gel particles were mounted on Cytoclear® slides and stored at −20°C. Microscopic images (1,388 × 1,040 pixels) of two filters per sample were captured at 200x magnification using a microscope (Axio Scope.A1, Zeiss) equipped with a camera (AxioCam MRc, Zeiss) using the Axio Vision LE64 Rel. 4.8 software (Zeiss). All particles bigger than 0.2 μm^2^ were analyzed. Further image analysis was conducted using ImageJ (Schneider et al., 2012). For TEP, sample images prepared with MilliQ water served as a blank, for CSP, the station and depth with the lowest concentration of CSP (station 1 at 400 m) served as a blank.

### Bacteria

Bacterial cell numbers were determined from a 4 ml sample that was fixed with 200 μl glutaraldehyde (GDA, 1% final concentration) and stored at −20°C for 2.5 months until analysis. Storing the samples for 2.5 months has been shown to underestimate bacterial abundances (Turley and Hughes, 1992), however, since all samples have been treated in the same way, comparability between samples is still warranted. SYBR Green I (Molecular Probes) was used to stain the samples. Bacterial abundance was determined with a flow cytometer (Becton and Dickinson FACScalibur) equipped with a 488 nm laser. The bacteria were detected using their unique signature in a plot of side scatter (SSC) vs. green fluorescence (FL1). Yellow-green latex beads (Polysciences, 0.5 μm) were measured as well as an internal standard.

A small number of TEP with the exact size of bacterial cells and extracellular DNA stuck to them could be counted by the flow cytometer as cells. However, most of the TEP will be outside the size range, i.e., too small or too large and will be discarded as background signal. Thus, even though some TEP might be counted as bacteria, their influence on the total number of bacteria is negligible.

### Phytoplankton

Since sampling volume is limited for the SML we used measurements of flow cytometry and phytoplankton absorption to assess the difference between SML and ULW which both require much less sampling volume than needed for HPLC pigment concentration. While the flow cytometry data only cover the composition of major groups for the pico- and nanophytoplankton fraction, the phytoplankton absorption data describe the whole phytoplankton community biomass (further details are provided below). HPLC pigment concentrations were only determined for adjacent surface waters (6 m depth).

#### Cell abundance of nano- and picophytoplankton

Phytoplankton cell numbers were determined from a 4 ml sample fixed with 200 μl GDA (1% final concentration) and stored at −20°C for 2.5 months until analysis. After filtering the samples through a 50 μm filter, the samples were analyzed using a flow cytometer (Becton & Dickinson FACScalibur) with a 488 nm laser and a standard filter set-up. A high flow rate (~39–41 μl min^−1^) was used to enumerate phytoplankton cells. The phytoplankton cells were distinguished based on their forward or right angle light scatter (FALS, RALS) as well as by their phycoerythrin and chl *a* related fluorescence signal. Cells within a size range of app. 0.2–20 μm diameter were detected, so no enumeration was obtained for microphytoplankton. Signals of very small picoplankton, such as the signal of *Prochlorococcus*, mixed with the background signal and could not be enumerated. Cell counts were analyzed with the help of the CellQuest Pro-Software (BD Biosciences).

#### Surface water total and major phytoplankton groups chlorophyll a concentration

Water from a continuous sea water supply of the ship (taken from ~6 m below the ship) was sampled. The samples were filtered onto GF/F filters (Whatman), shock-frozen in liquid nitrogen and stored at −80°C until analysis. The concentration of chl *a* was analyzed using a High Performance Liquid Chromatography (HPLC) technique according to Barlow et al. (1997), adjusted to the instrumentation used: pigment extracts were separated by a Waters HPLC system equipped with an autosampler (717 plus), pump (600) photo-diode array (2996), a fluorescence detector (2475), and the EMPOWER software (all Waters, Millipore).

Fifty microliters of an internal standard (canthaxanthin) and 1.5 ml acetone were added to each filter sample and then homogenized for 20 s in a Precellys© tissue homogenizer for analytical preparation. After centrifugation, the supernatant liquid was filtered through a 0.2 μm polytetra-fluoroethylene (PTFE) filter (Rotilabo) and placed in centrifuge tubes (Eppendorf). An aliquot of 100 μl was transferred to the autosampler (4°C). Just prior to analysis, the sample was premixed with 1 M ammonium acetate solution in the ratio 1:1 (v/v) in the autosampler and injected onto the HPLC system. The pigments were analyzed by reverse-phase HPLC, using a VARIAN Microsorb-MV3 C8 column (4.6 × 100 mm) and HPLC-grade solvents (Merck). Solvent A consisted of 70% methanol and 30% 1 mol l^−1^ ammonium acetate and solvent B contained 100% methanol. The gradient was modified after Barlow et al. (1997). Eluting pigments were detected by absorbance (440 nm). We determined the list of pigments shown in Table 1 of Taylor et al. (2011) and applied the method of Aiken et al. (2009) for quality control of the pigment data. Total chl *a* concentrations were calculated from the sum of the pigment concentrations of monovinyl chl *a*, divinyl chl *a*, and chlorophyllide *a*.

**Table 1:** Information on the stations of SO243 with latitude and longitude, sampled SML depth, and total station depth.

**SML station**	**Regime**	**Latitude [°]**	**Longitude [°]**	**SML thickness [μm]**	**Station depth [m]**	**UV A [W m^−2^]**	**PAR [W m^−2^]**	**Wind speed [m s^−1^]**	**Water temp [°C]**
1	Open	0.00	−85.50	39.23	3,200	42.11	360.73	9.8	24.9
2	Open	−2.50	−85.50	41.42	3,200	46.84	396.24	8.1	24.3
3	Open	−10.00	−81.92	41.83	4,500	22.87	192.81	9.8	20.7
4	Coastal	−9.00	−78.90	43.34	60	11.21	87.84	7.1	20.2
5	Coastal	−12.25	−77.08	40.67	80	27.73	224.90	3.8	17.6
6	Coastal	−12.36	−77.44	37.69	250	11.10	94.55	8.6	19.4
7	Upwelling	−14.40	−77.28	37.09	5,000	10.51	78.03	6.0	17.1
8	Upwelling	−14.04	−76.53	45.90	270	5.33	45.37	7.5	16.4
9	Upwelling	−16.07	−76.58	41.46	3,000	18.66	154.37	6.0	18.2
10	Upwelling	−15.67	−75.90	40.34	5,000	2.88	25.11	8.5	18.0
11	Upwelling	−15.32	−75.27	45.65	120	12.88	100.00	7.5	15.9

The chl *a* concentrations of the major phytoplankton groups explaining the total phytoplankton biomass were calculated following the diagnostic pigment analysis developed by Vidussi et al. (2001) and adapted in Uitz et al. (2006) to relate the weighted sum of seven, for each phytoplankton group representative diagnostic pigments (DP). By that the chl *a* concentrations (conc.) for diatoms, dinoflagellates, haptophytes, chrysophytes, cryptophytes, cyanobacteria (excluding prochlorophytes), and chlorophytes were derived. The chl *a* conc. of prochlorophytes was derived directly from the divinyl chl *a* conc. (the marker pigment for this group).

#### Dissolved organic matter and phytoplankton absorption in the SML and ULW

The light absorption coefficient spectra of the chromophoric dissolved organic matter (CDOM) fraction and the particulate fraction from samples of the SML and ULW were determined. The particulate fraction (0.1–0.5 l) was collected by filtration onto GF/F (Whatman) filters, while the dissolved fraction was additionally filtered through 0.2 μm membrane material.

The spectral absorption coefficient of the CDOM was determined using a liquid waveguide capillary cell setup (LWCC, WPI) as described in Röttgers and Koch (2012), with a 50 cm and a 250 cm path length cell. Resulting spectra were corrected for salinity effects using concomitant measurements to determine the attenuation of a NaCl solution to mimic optical effects of seawater. Absorption coefficients of all particles (phytoplanktonic and non-phytoplanktonic) are determined by the quantitative filter technique (QFT) using an integrating-cavity absorption-meter setup (QFT-ICAM) as described by Röttgers et al. (2016). Non-phytoplankton absorption coefficients are determined in the same way after the algal pigments have been removed by oxidative bleaching with a few drops of a NaOCl solution (1% active Cl), and after a few minutes adding a few drops of a 1% H_2_O_2_ solution to oxidize the remaining NaOCl. Phytoplankton absorption coefficients were calculated by subtracting non-phytoplankton particle absorption from the total particulate absorption. The phytoplankton absorption at 670 nm (that of “living” chl *a*) is used as a robust indicator for phytoplankton biomass (Bricaud et al., 1995); as direct chl *a* concentrations from the SML and ULW are not available.

### Irradiance measurements

Continuous solar irradiance was measured using a RAMSES hyperspectral radiometer (TriOS GmbH, Germany), in the wavelength range from 350 to 950 nm and with a spectral resolution of ~3.3 nm and a spectral accuracy of 0.3 nm. All measurements were obtained with an automatically set integration time of the respective sensor (4 ms and 8 s) which were then averaged over 10 min. PAR irradiance was calculated as the integral of irradiances for wavelengths from 400 to 700 nm for each measurement, and the UVA irradiance was calculated as the integral for wavelengths from 320 to 400 nm (unfortunately, sensor calibration was not optimal and the measurements from 320 to 350 nm are questionable).

### Data obtained from the ship

Abiotic data was obtained from the DSHIP system onboard RV *Sonne*. Wind speed was measured at the top of the ship, 35 m above the sea surface. The water temperature was measured below the ship at a depth of 2 m.

### Data analysis

The enrichment factor, EF, was calculated to determine the concentration difference of substance A in the SML compared to the ULW.

(2) $$\begin{array}{l} \text{EF }=\;{\left[\text{A}\right]}_{\text{SML}}/{\left[\text{A}\right]}_{\text{ULW}} \end{array}$$

where [A] is the concentration of a certain substance in the SML or the ULW (Unesco, 1995). An EF > 1 indicates an enrichment of a certain substance, whereas an EF < 1 indicates lower concentrations of a certain substance in the SML compared to the ULW.

The statistical analyses were conducted in the R statistical environment (R Development Core Team, 2013). The data was checked for normality using the Shapiro-Wilk-test (*n* = 11, α = 0.05). The test showed that the data was not normally distributed (*n* = 11, α = 0.05). Thus, the Mann-Whitney *U*-test was used for comparison of the SML and the ULW (*n* = 11, α = 0.05) and the Kruskal-Wallis test was used for comparing the three regimes (*n* = 6–10, α = 0.05, *p*-values corrected for 3-way comparison).

The organic matter composition of the SML and the ULW was used to compute a dendrogram of the differences between stations (parameters included in calculations: TEP abundance, TEP area, CSP abundance, CSP area, TOC, DOC, TN, TDN, TCHO, DCHO, TAA, DAA, FAA). To compute the dendrogram, the dissimilarity matrix was calculated using Euclidean distance (using the root of the sum-of-squares of the differences between samples). For the calculation the data were standardized (mean value subtracted, divided by mean absolute deviation) to enable an equal contribution of parameters with different concentrations. The samples were clustered by their averages. The dendrogram was computed and visualized using the R packages *ggdendro* and *ggplot2* (Wickham, 2009; de Vries and Ripley, 2016).

Microsoft Office Excel 2010 was used for data analysis. Ocean Data View was used to create maps (Schlitzer, 2013).

## Results

### Hydrographic conditions and overall phytoplankton abundance and composition in the surface water

The wind speed during the SO243 cruise ranged from 3.8 to 9.8 m s^−1^. Except at station 5 with 3.8 m s^−1^, at all other stations wind speeds were >6 m s^−1^ (Table 1). The sea surface temperature at ~2 m depth varied between 16 and 25°C between the first and the last station (station 1 and 11). Based on HPLC marker pigment concentrations at 6 m water depth during the whole cruise, diatoms, and haptophytes showed the highest chl *a* concentrations (indicator of biomass) with on average 234 and 276 ng chl *a* l^−1^ (maxima of 965 and 407 ng chl *a* l^−1^, respectively) among all phytoplankton groups. Dinoflagellates, cyanobacteria, chlorophytes, and chrysophytes occurred in medium concentrations with average concentrations of 139, 88, 53, and 31 ng chl *a* l^−1^ and maxima of 332, 113, 175, 48 ng chl *a* l^−1^, respectively. Compared to these phytoplankton groups, cryptophytes and prochlorophytes occurred in rather low concentrations (average 4 and 11, maxima 9 and 43 ng chl *a* l^−1^).

In order to compare different regions with regard to the enrichment and composition of organic matter and microorganisms in the SML, we classified three different regimes: (I) open ocean, (II) coastal stations, and (III) upwelling stations. The 11 stations are divided into these regimes as shown in Figure 1.

**Figure 2:**
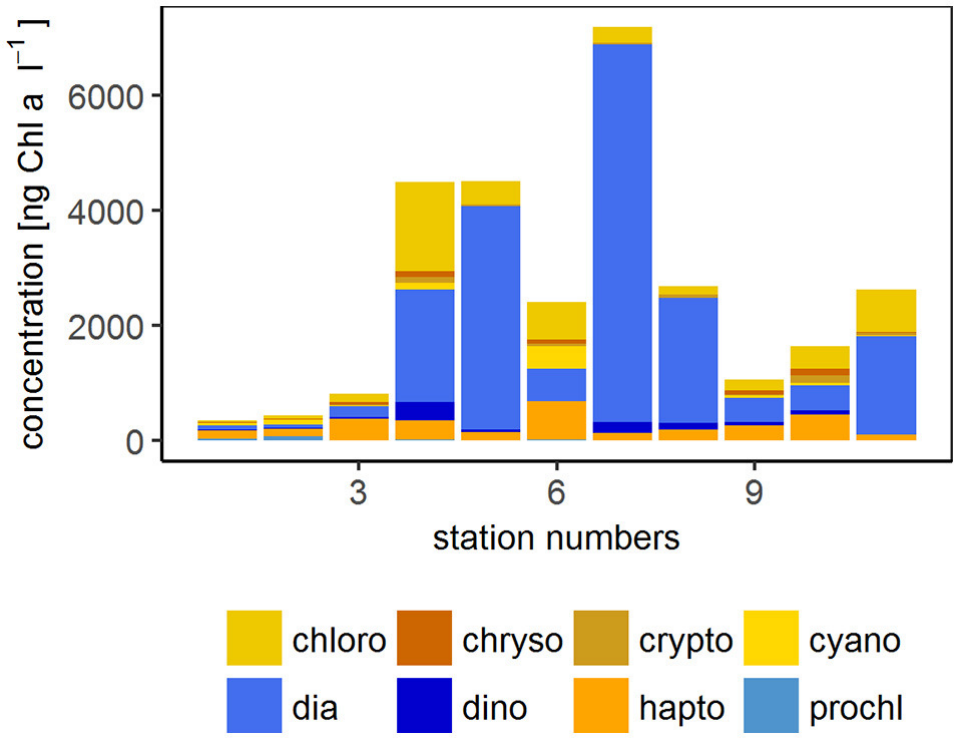
Phytoplankton community composition at 6 m during SO243. The concentration of the different phytoplankton groups are derived from HPLC measurements. chloro, Chlorophytes; chryso, Chrysophytes; crypto, Cryptophytes; cyano, Cyanobacteria; dia, Diatoms; dino, Dinoflagellates; hapto, Haptophytes; prochl, Prochlorococcus. Blue colors indicate groups that were not detected with the flow cytometer (diatoms, dinoflagellates, and Prochlorococcus).

The open ocean stations were comprised of stations 1–3 and were located off the Peruvian shelf. High wind speeds (8.1–9.8 m s^−1^) occurred in the open ocean regime. The phytoplankton biomass was rather low at the open ocean sites (354–807 ng chl *a* l^−1^ with an average of 532 ng chl *a* l^−1^). The contribution of haptophytes with 30–50% was the largest, followed by diatoms (15–22%), and then chlorophytes, cyanobacteria, and prochlorophytes (each around 10%) (Figure 2). UVA and PAR irradiance was high at stations S1 and S2 resulting in a higher average irradiation in the open ocean regime than in the other two regimes which were further south (Table 1).

The coastal stations comprised stations 4–6. The stations were located on the shelf within 50 km of the coast in the vicinity of two major cities (Chimbote and Lima) and showed high chl *a* concentrations (2,409–4,509 ng chl *a* l^−1^ with an average of 3,807 ng chl *a* l^−1^). The coastal regime had the highest biomass of diatoms (contributing 23–86%), chlorophytes (10–35%), dinoflagellates, and haptophytes (3–46%, inversely related to diatom fractions), while cyanobacteria were only contributing 17% at station 6 (absent at the other stations) (Figure 2). These stations were also influenced by El Niño conditions which resulted in slightly reduced upwelling indicated by low nutrient N:P ratios in the upper 150 m and higher water temperature, salinity, and oxygen concentrations below 50 m (Stramma et al., 2016).

Stations 7–11 represented the upwelling stations influenced by cold, high salinity water. Medium to very high chl *a* concentrations (1,060–7,195 ng chl *a* l^−1^ with an average of 2,921 ng chl *a* l^−1^) were found in the upwelling regime. In the upwelling system, diatoms dominated the majority of the stations (contributing 26–91%) with similar concentrations of haptophytes and chlorophytes (2–28 and 4–28%, respectively) and only minor contributions <10% from dinoflagellates, crysophytes, cryptophytes, and cyanobacteria (Figure 2). The effect of El Niño decreased from north to south (Stramma et al., 2016) with a low influence on the stations in the upwelling region. These stations had lower surface water temperatures between 14 and 17°S than the other two regimes (Figure 1) (Stramma et al., 2016).

### Concentration and accumulation of organic matter in the SML

The thickness of the surface layer varied between 37 and 46 μm with a mean of 41.3 ± 2.7 μm (*n* = 11), which is in good accordance with literature values for glass plate sampling (Cunliffe et al., 2011, 2013; Engel and Galgani, 2016). The SML thickness was not related to wind speed, water temperature, or the peak of the phytoplankton absorption coefficient at 670 nm (*a*_ph_670) representing the phytoplankton biomass.

General bacterial abundances in the SML and the ULW ranged between 4.42 × 10^5^ and 1.89 × 10^6^ cells ml^−1^ with a mean value of 9.03 × 10^5^ ± 4.48 × 10^5^ cells ml^−1^.

Three different groups of nano- and pico-phytoplankton were distinguished: (I) non-cyanobacteria like phytoplankton (NCBL) containing only chl *a*, (II) small cyanobacteria like cells (pico-CBL) containing phycoerythrin and chl *a*, and (III) larger cells containing phycoerythrin and chl *a*, labeled as TrL (*Trichodesmium*-like cells). Further analyses to determine the taxonomy of the phytoplankton in more detail were not conducted. The pico-CBL abundance correlated with the cyanobacterial chl *a* concentration determined by HPLC at 6 m (rho = 0.71, *p* = 0.01).

Total nano- and pico-phytoplankton abundance ranged from 2.32 × 10^3^ to 2.92 × 10^4^ cells ml^−1^ with an average abundance of 7.49 × 10^3^ ± 7.18 × 10^3^ cells ml^−1^ (TrL 778 ± 387 cells ml^−1^, pico-CBL 437 ± 486 cells ml^−1^, and NCBL 6.27 × 10^3^ ± 6.67 × 10^3^ cells ml^−1^).

TOC concentrations ranged from 66 to 116 μmol l^−1^ with an average of 82 ± 13 μmol l^−1^. DOC concentrations were slightly lower and ranged from 57 to 111 μmol l^−1^ with an average of 76 ± 14 μmol l^−1^. TN and TDN concentrations varied between 11 and 33 μmol l^−1^ and 11 and 31 μmol l^−1^, respectively with averages at 23 ± 6 and 22 ± 6 μmol l^−1^, respectively. Particulate nitrogen (PN) concentrations calculated as TDN subtracted from TN, ranged from 0.2 to 3.5 μmol l^−1^ with an average of 1.1 ± 0.9 μmol l^−1^.

TCHO concentrations varied between 463 and 1,495 nmol l^−1^. The average TCHO concentration was 738 ± 275 nmol l^−1^. DCHO concentrations were clearly lower ranging from 288 to 1,200 nmol l^−1^ with an average of 509 ± 175 nmol l^−1^. The concentration of TAA ranged between 364 and 2,868 nmol l^−1^, with an average of 1,171 ± 662 nmol l^−1^, while DAA concentrations ranged between 202 to 2,007 nmol l^−1^ with an average of 594 ± 549 nmol l ^−1^. FAA concentrations were distinctly lower ranging from 32 to 1,268 nmol l^−1^ with an average of 298 ± 320 nmol l^−1^.

CSP were found in higher abundances from 6.23 × 10^5^ to 2.15 × 10^7^ particles l^−1^ (average of 1.06 × 10^7^ ± 5.58 × 10^6^ particles l^−1^) than TEP which ranged from 5.78 × 10^5^ to 1.18 × 10^7^ particles l^−1^ (average of 5.16 × 10^6^ ± 3.04 × 10^6^ particles l^−1^). Consistent with this trend, the total CSP area was higher than the total TEP area (7.73 × 10^7^ ± 4.77 × 10^7^ compared to 2.58 × 10^7^ ± 1.95 × 10^7^ μm^2^ l^−1^).

**Figure 3:**
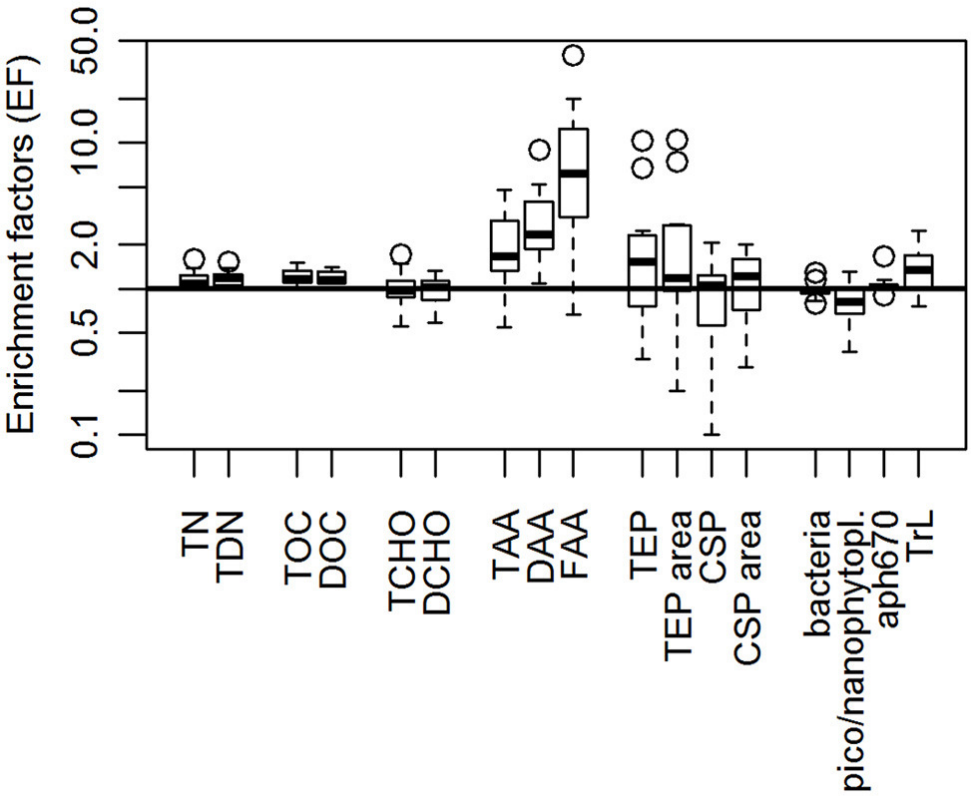
Enrichment factors (EFs) of organic components in the SML during SO243. T/TDN, total/total dissolved nitrogen; T/DOC, total/dissolved organic carbon; T/DCHO, total/dissolved hydrolysable carbohydrates; T/D/FAA, total/dissolved/free amino acids; TEP, Transparent Exopolymer Particles; CSP, Coomassie Stainable Particles; pico/nanophytopl, pico-/nanophytoplankton abundance derived from flow cytometry; aph670, phytoplankton absorption measurements at 670 nm (as proxy for chlorophyll a concentration); TrL, Trichodesmium-like cells derived from flow cytometry. Boxplots are derived from 11 stations each. Outliers are displayed as open circles.

Figure 3 indicates the enrichment of organic matter in the SML. Enrichment factors (EF) of TOC and DOC varied between 1.1–1.4 and 1.0–1.5, respectively. TCHO and DCHO had varying EF of 0.6–1.7 and 0.6–1.3, respectively. While DAA were at least slightly enriched in the SML at all stations (EF = 1.1–9.0), TAA and FAA were depleted at station 11 (both with an EF = 0.6 at station 11) but showed an enrichment at all other stations.

The gel particles, TEP and CSP, showed different trends with regard to their enrichment in the SML. TEP seemed to be enriched in the SML with respect to both abundance and total area, whereas the abundance of CSP was not different in the SML and ULW. Total CSP area however seemed to be marginally higher in the SML (Figure 3) indicating prevalence of larger CSP in the SML.

Bacterial numbers were overall slightly reduced in the SML. The enrichment factor ranged from 0.8 to 1.3, with a mean of 1.0. The trend of *a*_ph_670 showed a similar behavior with a mean EF of 1.1 ± 0.2. Flow-cytometry measurements showed that NCBL and pico-CBL showed similar trends (0.3–1.3 with a mean of 0.8; 0.5–1.0 with a mean of 0.8, respectively). TrL however were mostly enriched in the SML with a mean enrichment of 1.5 (ranging from 0.8–2.5). Correlations of SML and ULW concentrations of the measured parameters were listed in Table 2.

**Table 2:** Correlations of SO243 microbiological and organic parameters.

**Parameter**	**ULW**	**Global rad**	**Wind speed**	**Surf temp**	**Surf chl *a***
Bacteria	0.94	–	–	–	0.62
NCLP	0.84	–	–	–	0.65
Pico-CBL	0.97	-	–	0.75	–
*TrL*	–	–	–	–	–
Phyto-abs	0.97	–	−0.70	−0.63	0.94
TEP	–	–	–	–	–
TEP area	–	–	–	–	–
CSP	–	–	–	–	–
CSP area	–	–	–	–	–
TOC	–	0.76	–	0.64	–
DOC	0.79	0.86	–	0.78	–
TCHO	–	–	–	–	–
DCHO	–	0.80	–	–	–
TAA	–	–	–	–	–
DAA	–	–	–	–	–
FAA	–	0.71	–	–	–
TN	0.87	–	–	–	0.72
TDN	0.83	–	−0.60	–	0.75

*ULW: Correlations between the concentrations in the SML and the ULW. All other columns: Correlations between the concentrations of the specific parameter in the SML and ULW combined with abiotic factors such as global radiation, wind speed, surface temperature and surface chlorophyll a concentrations. All correlations shown are presented as rho-values and were calculated using Spearman correlation and have a p-value < 0.05 (n = 11)*.

### Differences in the spatial distribution

As outlined above, three different regimes were distinguished according to their location, chl *a* concentration and the influence of upwelling: the open ocean regime (S1-3), the coastal regime (S4-6), and the upwelling regime (S7-11).

**Figure 4:**
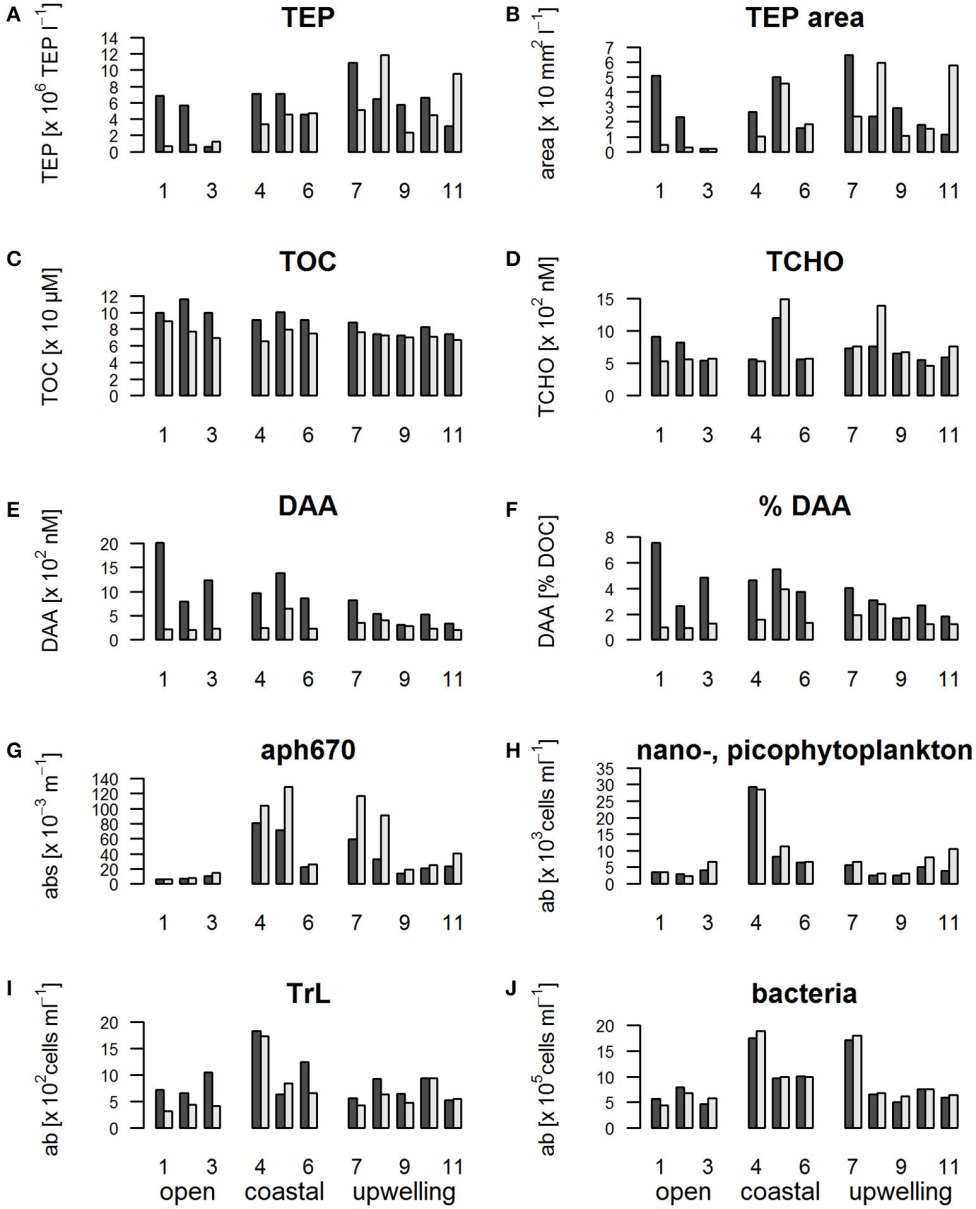
Barplots showing the concentration of organic matter and microorganisms in the SML compared to the ULW during SO243 off Peru; black: SML, white: ULW. **(A)** Abundance of TEP (Transparent Exopolymer Particles), **(B)** total TEP area, **(C)** TOC (total organic carbon), **(D)** TCHO (total hydrolysable carbohydrates), **(E)** DAA (dissolved amino acids), **(F)** %DAA (% C of DOC that is made up of DAA), **(G)** aph670 (phytoplankton absorption at 670 nm as a proxy for chlorophyll a concentration), **(H)** nano-picophytoplankton abundance derived from flow cytometry measurements, **(I)** TrL abundance, **(J)** bacterial abundance.

Bacteria were found in similar abundances at upwelling and coastal stations with highest numbers at stations 4 and 7 (Figure 4J). Bacteria were significantly more abundant in the coastal regime compared to the open ocean regime (Kruskal-Wallis test, *p* = 0.015). However, there were no significant differences in the bacterial abundances between the SML and the ULW (Table 3).

**Table 3:** Significant differences in the distribution of parameters between the SML and the ULW (ULW) and between different regimes (regime).

**Parameter**	**ULW**	**Regime**
TOC	<0.01	–
DOC	<0.05	<0.05
TCHO	–	–
DCHO	–	–
TAA	<0.05	–
DAA	<0.001	–
FAA	<0.001	–
TN	–	<0.05
TDN	–	<0.05
Bacteria	–	<0.01
NCBL	–	<0.01
Pico-CBL	–	<0.01
Phyto-abs	–	<0.01
TrL	<0.05	–
TEP	–	–
CSP	–	–

*The differences at depth were calculated using the Mann-Whitney U-test, the differences between regimes with the Kruskal-Wallis-test. P-values of significant differences are shown (n = 11). For the Kruskal-Wallis-test p-values were corrected for multiple comparisons*.

In general the values of *a*_ph_670 showed no significant difference between the SML and ULW. The *a*_ph_670 was lowest in the open ocean stations, significantly lower than in the coastal or upwelling stations. However, the two highest enrichments were also observed in the open ocean regime at stations 1 and 2 (Figure 4G). No significant differences were detected between the coastal and the upwelling regime in terms of *a*_ph_670.

By flow cytometry, phytoplankton cells between 0.2 and 20 μm diameter—which includes the pico- and nano-phytoplankton fraction were further distinguished (Figure 4H). The three identified phytoplankton groups differed with respect to their distribution patterns. NCBL and pico-CBL did not show differences in their abundances between SML and ULW (Table 3). They did however show differences in their numbers between regimes. The abundance of pico-CBL in the upwelling stations was significantly lower than in the open ocean or the coastal regime. NCBL on the other hand showed their highest abundance in the coastal stations. Their abundance in the open ocean and at the upwelling stations was similar (data not shown).

**Figure 5:**
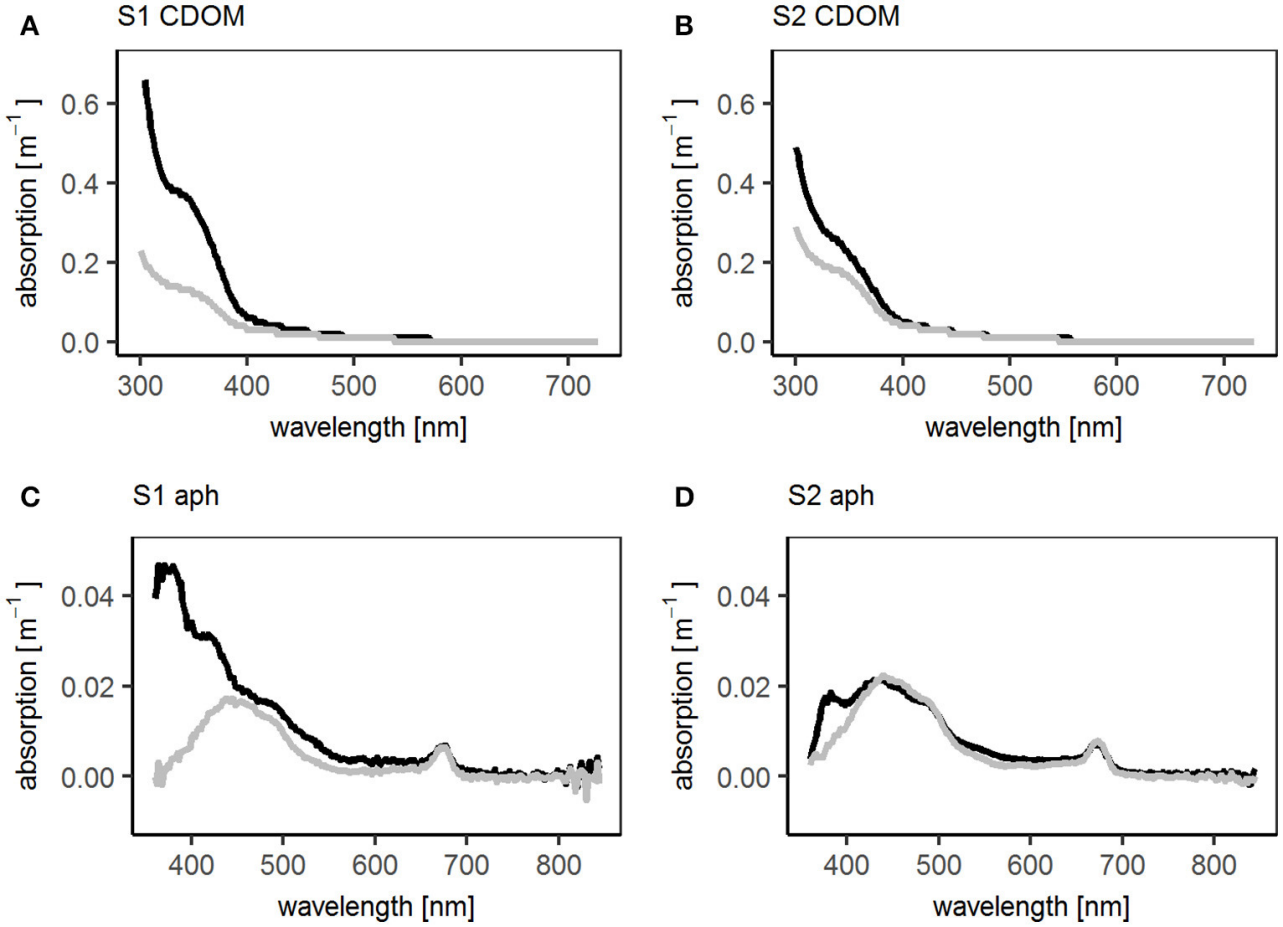
Absorption spectra of CDOM (chromophoric dissolved organic matter) and aph (phytoplankton absorption) at SO243 stations S1 and S2. Black: in SML, gray: in ULW. **(A)** Absorption spectra of CDOM at station S1, **(B)** absorption spectra of CDOM at station S2, **(C)** absorption spectra of phytoplankton at station S1, **(D)** absorption spectra of phytoplankton at station S2.

The third phytoplankton group, the TrL, had a different distribution to the other two phytoplankton groups. Despite showing no differences in their horizontal distribution, the TrL were often enriched in the SML (Figure 4I). The enrichment was most pronounced in the open ocean stations, while at one station (station 5) in the coastal regime a slight decrease of TrL abundance in the SML was observed. In the upwelling regime, the TrL were either enriched or had the same concentration in the SML compared to the ULW. The abundance of TrL corresponded to the increase of the phytoplankton absorption peak at 360 nm, indicating the presence of MAAs (Uemura et al., 1980; Dupouy et al., 2008). This peak was especially pronounced at stations 1 and 2 (Figure 5) where also UVA radiation was high.

The overall trend of CSP area followed the same trend as for CSP abundance, with no significant changes between the regimes. CSP tended to be enriched in the SML in the open and coastal stations, while it showed a decreasing trend in the SML of the upwelling stations (data not shown). However, those differences with depth were not significant.

TEP abundance in the ULW in the open regime was lowest of all stations (Figure 4A). High TEP abundances were still found in the SML of the open stations though, indicating strong TEP enrichment at the open ocean stations despite the high wind speed. In the upwelling regime the enrichment of TEP in the SML was less pronounced, with some stations showing an enrichment and others a reduction in TEP abundance compared to the ULW (Figure 4A).

Stations 1 and 2 stood out regarding the enrichment of TEP (Figures 4A,B) in the SML (10.4 and 6.7 repectively compared to an overall mean EF of 2.7). High UVA and PAR (42,107 and 46,838 W m^−2^ UV A; 360,729 and 396,238 W m^−2^ PAR) were observed at both stations.

TOC (Figure 4C), as well as DOC concentrations (data not shown) in the ULW were similar between different regimes. In the SML, the concentration of TOC and DOC was decreasing from the open stations, to the coastal and upwelling regimes. In the same way the enrichment in the SML of TOC and DOC decreased slightly from the open stations to the upwelling regime.

Like TOC and DOC, TCHO and DCHO concentrations were rather similar across the three regimes (Figure 4D) with DCHO (data not shown) showing the same trends as TCHO. No clear differences in the enrichment of TCHO and DCHO between the regimes were observed. However, stations 1 and 2 with the high radiation had the highest enrichment of TCHO (1.5 and 1.2 respectively, compared to an overall mean of 1.0). For DCHO an EF of 1.2 was detected at stations 1 and 2. These EFs were high compared with other stations, not the highest recorded during this study for DCHO though. In addition, only stations 1 and 2 showed peaks in the absorption spectra of the CDOM in the SML and ULW at 330 nm indicating the presence of MAAs (Figure 5) (Dupouy et al., 2008). At both stations the 330 nm peak was higher in the SML than in the ULW, though the difference was more pronounced at station S1.

All amino acid fractions (TAA, DAA, and FAA) followed the same trend (TAA/FAA data not shown) with a particularly high enrichment in the SML at the open ocean sites (Figure 4E) with EFs of 1.4, 3.9, and 4.7 for stations 1–3 compared to an average of 1.7 for TAA, EFs of 9.0, 3.9, 5.2 for stations 1–3 with an overall cruise average of 2.3 for DAA and EFs of 39.9, 12.8, and 20.2 compared to an overall cruise average of 6.2 for FAA. Their enrichment decreased from the open to the coastal to the upwelling regime.

The DAA enrichment decreased from the open to the coastal to the upwelling regime. In the same manner, the percentage of DOC that is made up of carbon from DAA (Figure 4F) decreased from the open to the coastal to the upwelling regime in the SML. The higher the percentage of DAA, the less degraded the DOM is (Amon et al., 2001; Davis et al., 2009). Thus, in the SML of the open ocean, the DOM was the least degraded. The DOM degradation state in the SML increased toward the coastal region and was highest at the upwelling stations.

### The SML as a distinct habitat

**Figure 6:**
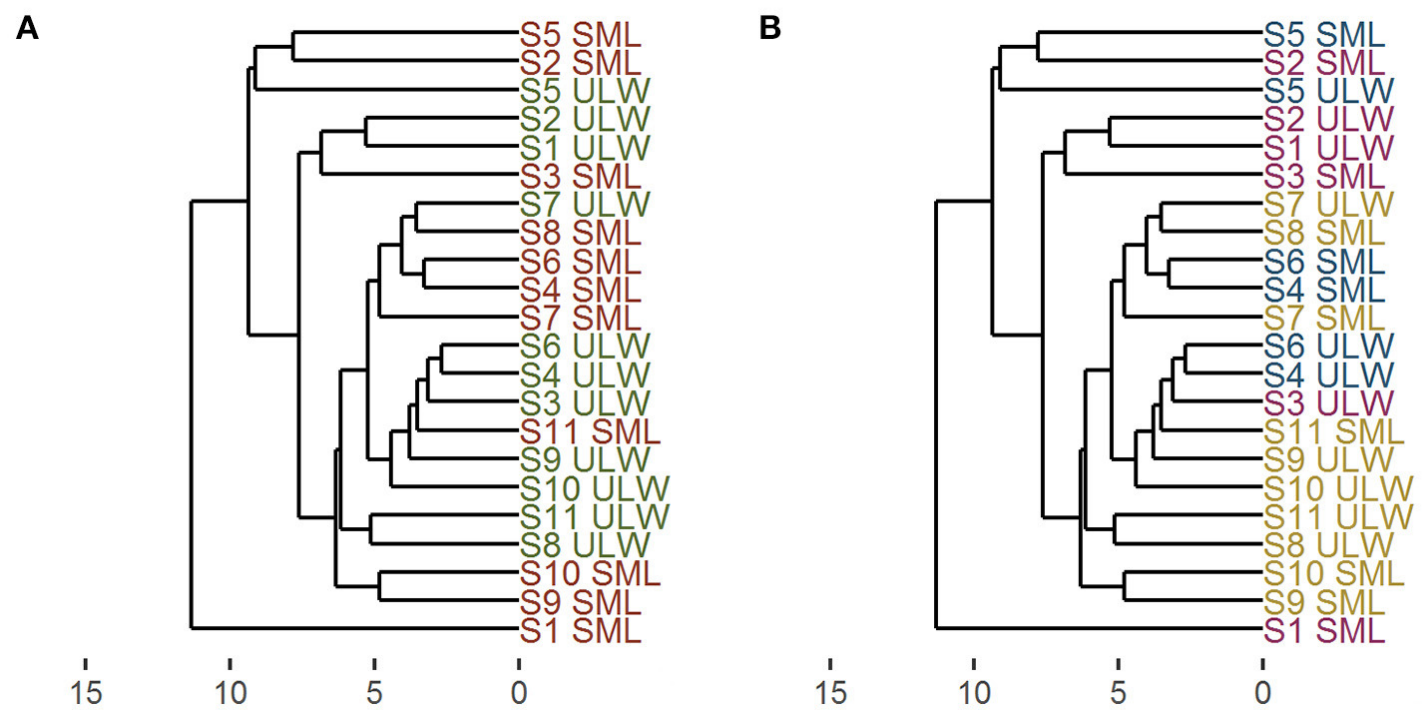
Dendrogram derived from cluster analysis using Euclidean distance and UPGMA (unweighted paired group method with arithmetic mean). All organic parameters were used to calculate the dendrogram. Numbers indicate station numbers, SML, surface microlayer; ULW, underlying water. X-axis: dissimilarity between stations. **(A)** Comparison of SML and ULW–SML: red, ULW: green; **(B)** comparison of different regimes—open stations: purple, coastal stations: blue, upwelling stations: yellow.

The similarity of all stations with regard to their organic composition (T/DOC, T/DCHO, T/D/FAA, T/TDN, TEP, CSP) as indicated from their Euclidean distance, is displayed in Figure 6. At all stations, the specific ULW showed more similarity to the ULW of other stations than to the SML of that specific station, and vice versa. The SML at station 1, one of the stations with the highest irradiance, seemed to be quite different in its organic matter composition from the rest of the stations, the SML at station 2 on the other hand which also had high irradiance values, was quite similar to the SML of station 5. The ULW at stations S1 and S2 were similar, while the SML composition seemed to be quite different.

Figure 6B shows the station names color-coded according to the different regimes. The results suggest no clear trend in the overall organic matter composition between the different regimes.

## Discussion

Numerous studies have investigated the microbial community and organic matter composition of the SML in different habitats such as the Mediterranean Sea, tropical regions or the Arctic (Agogué et al., 2005a; Leck and Bigg, 2005; Matrai et al., 2008; Engel and Galgani, 2016; Galgani and Engel, 2016; Galgani et al., 2016). However, most studies concentrated on one habitat or compared slick to non-slick conditions of the SML. This study aims at investigating the differences in organic matter enrichment in the SML in different systems off the Peruvian coast influenced by varying phytoplankton biomass (indicated by chl *a* concentrations) and location with regard to the continental shelf and distance from the coast as well as upwelling.

### Concentrations of organic matter and organisms in the peruvian upwelling region

The consistent enrichment of DOM in the SML compared to the ULW that we found is in good accordance with previous reports from the Peruvian upwelling system (Engel and Galgani, 2016) and has been observed previously also in other oceanic systems (Reinthaler et al., 2008; Wurl and Holmes, 2008).

Three years prior to this study, the cruise M91 with the RV *Meteor* also headed to the Peruvian upwelling region slightly later in the year in December 2012. During M91, organic matter parameters such as organic carbon, carbohydrates, and amino acids were sampled (Engel and Galgani, 2016). The location of the stations was comparable to that of the here presented study (from SO243). However, there was no El Niño event in 2012. The wind speed was higher with on average 7.5 ± 1.7 m s^−1^ during SO243 compared to 5.7 ± 2.1 m s^−1^ during M91.

Compared to what was previously found during M91 the range of DOC is similar, while during SO243 TOC concentrations were lower (66–116 μmol l^−1^ compared to 82–199 μmol l^−1^) (Engel and Galgani, 2016). Furthermore, the DCHO concentrations were about half of those previously found (509 ± 175 nmol l^−1^ compared to 1,111 ± 550 nmol l^−1^) (Engel and Galgani, 2016). Also the TEP area was smaller (1.9–64.7 mm^2^ l^−1^ in comparison to 6.9–408 mm^2^ l^−1^), while the CSP area was comparable (Engel and Galgani, 2016).

TEP are operationally defined as gel particles containing acidic polysaccharides (Mopper et al., 1995). Thus, if DCHO concentrations were lower during SO243, it seems plausible that the area and abundance of TEP were also lower. When there are fewer precursors for TEP formation, then less TEP and possibly smaller TEP will form. As TOC concentrations are influenced by TCHO, a lower exudation of carbohydrates by phytoplankton would also decrease the TOC pool.

While bacterial numbers were similar to those of M91, the nano- and picophytoplankton cell numbers were lower (Engel and Galgani, 2016). Considering that phytoplankton exudates are one of the main sources for TEP precursors (Chin et al., 1998; Passow, 2002; Engel et al., 2004; Verdugo et al., 2004), the low area and abundance of TEP could be a result of lower nano- and picophytoplankton cell numbers and thus less production of TEP precursors such as DCHO. During SO243, the overall phytoplankton biomass trend (indicated by phytoplankton absorption at 670 nm) from ULW to SML was following the trend of the nano- and picophytoplankton cell numbers. However for M91, one has to keep in mind that the total phytoplankton biomass was not assessed, and it is not clear whether the concentration of these smaller phytoplankton groups were following that of the overall phytoplankton.

Concentrations of about 3.3 μmol l^−1^ particulate nitrogen were measured during M91 (Engel and Galgani, 2016) while during SO243 they were about 1.1 μmol l^−1^. However, the pico and nano-phytoplankton cell number concentrations were much higher (45 × 10^3^ ml^−1^) during M91 compared to 7.5 cells × 10^3^ ml^−1^ measured during our campaign. Thus, the sharp decrease in phytoplankton number concentrations during SO243 might explain why we found much lower particulate nitrogen compared to dissolved nitrogen concentrations.

The enrichment of the different organic matter components, including TEP area and DCHO, were similar to what has been previously found in the Peruvian upwelling region (Engel and Galgani, 2016) suggesting that the same transport as well as production and degradation processes between the SML and the ULW occurred despite El Niño (Stramma et al., 2016). However, the overall lower concentrations of organic matter, including organic carbon, carbohydrates, and TEP indicate a lower primary production in October 2015 compared to December 2012. The lower organic matter concentrations are in accordance with lower silicate and phosphate concentrations in the surface ocean in October 2015 compared to December 2012 (Stramma et al., 2016) which could explain why potentially the primary productivity was lower in 2015.

### The SML as a distinct and variable habitat and its impact

As outlined above, three different regimes were distinguished according to their location, chl *a* concentration and the influence of upwelling. The first regime, the open ocean regime (S1-3), was characterized by water not in the upwelling region and off the Peruvian shelf as well as by low chl *a* concentrations. The second regime, the coastal stations (S4-6), was characterized by its proximity to Chimbote and Lima, two big cities with a lot of ship traffic, discharge from land and industry, and thus a potentially high anthropogenic impact (Cury et al., 2011; Castro and Fillmann, 2012; Capone and Hutchins, 2013). The chl *a* concentrations were highest in the coastal regime. The upwelling regime contained stations that were located directly in the upwelling (S7-11) (Stramma et al., 2016) and that were less impacted by El Niño as the influence decreased from the northern to the southern stations.

Different trends in the enrichment of microorganisms within the SML could be observed during this study. The differences between regimes were tested for the SML and ULW samples together. Significant differences were observed for bacteria, NCBL and pico-CBL. Only the TrL did not show significant differences between regimes. Bacteria and NCBL showed their lowest abundance in the open stations which may be explained by potentially lower productivity expected for these stations. The station with the highest abundance of bacteria, pico-CBL, and NCBL was located in the coastal regime, suggesting that the microorganisms were limited in the open and upwelling stations. Limiting factors could have been e.g., nutrients or DOM concentrations.

It has been suggested that microorganisms in the SML are mainly recruited from the ULW (Agogué et al., 2005a; Obernosterer et al., 2008; Cunliffe et al., 2009; Stolle et al., 2011). It is often assumed that cells face adverse conditions in the SML like high solar radiation, which results in a decrease in bacterial abundances as well as in their activity and production (Aller et al., 2005; Joux et al., 2006; Stolle et al., 2010). In this study, bacterial numbers were depleted in the SML relative to the ULW in some stations, and enriched in others, with no clear correlations to irradiance, wind speed or surface chl *a* concentrations, suggesting that irradiance did not drive bacterial distribution directly.

In contrast to bacterial patterns, phytoplankton enrichment in the SML has been observed in various locations (Hardy and Apts, 1984, 1989; Joux et al., 2006). While the overall phytoplankton biomass determined by absorption showed no difference between SML and ULW and the phytoplankton cell abundance determined by flow cytometry in the SML was reduced at most stations (mean EF of 0.8), a different pattern was observed for single phytoplankton groups. While NCBL and pico-CBL showed a decreasing abundance in the SML, TrL were enriched.

Microorganisms can protect themselves from UV light by producing e.g., mycosporine-like amino acids (Tilstone et al., 2010). As mentioned before, TrL show a positive trend with the height of the phytoplankton absorption peak at 360 nm. An absorption peak at 360 nm has been identified as palythene, a MAA, in previous studies (Uemura et al., 1980; Dupouy et al., 2008). Palythene production has been observed amongst others in Cyanobacteria and Dinophyceae (Dupouy et al., 2008; Vale, 2015). *Trichodesmium* has been associated with two peaks, at 330 and 360 nm (Dupouy et al., 2008) which suggests that they produce MAAs as light protection mechanism. The highest enrichments of TrL have been found in the open ocean regime with the highest irradiation. At the same stations the highest peak of the dissolved and phytoplankton absorption at 330 and 360 nm, respectively, was detected in the SML and ULW, which further underlines the relationship between TrL and palythene. *Trichodesmium* has been detected in high abundances in the SML before (Nesterova, 1980; Hardy and Antrim, 1986) providing further evidence that TrL are mainly composed of *Trichodesmium*. The organisms are naturally buoyant due to gas vesicles allowing them to stay at the sea surface (Baalen and Brown, 1969; Gantt et al., 1984; Capone et al., 1997).

*Trichodesmium* belongs to the group of diazotrophic phytoplankton and their nitrogen fixation has been suggested to play an important part in the marine nitrogen cycle (Capone et al., 1997). Therefore, the enrichment of the diazotrophic *Trichodesmium* could provide an explanation for the generally elevated total and dissolved nitrogen concentrations found throughout the cruise in the SML compared to the ULW. An indication for TrL encountering favorable conditions in the SML is the fact that even though there were no clear differences in the concentration of TrL between regimes, a difference in the enrichment could be observed. While TrL were clearly enriched in the SML of the open stations, only a slight enrichment was observed in the coastal regime and only slight or no enrichment in the upwelling regime. This suggests that the enrichment of TrL in the SML is driven by the availability of organic matter and nutrients in the SML, which tend to be enriched (Agogué et al., 2005a; Cunliffe et al., 2013). The high radiation might inhibit other phytoplankton species which are unable to produce sufficient MAAs and might enable *Trichodesmium* to dominate the phytoplankton community in the SML. Thus, in the most nutrient-depleted environment, the open ocean stations, TrL seem to move up in the water column where organic material and nutrients are enriched. The overall concentrations of TrL in the open ocean stations were still lower than in the coastal stations though, suggesting that they were growth limited. Even though we found a lot of evidence in our study pointing toward *Trichodesmium*, further DNA investigations would be necessary to undoubtedly determine whether TrL are mainly composed of *Trichodesmium*.

Even higher differences in the SML enrichment trends were observed for the gel particles. The pattern of the area and the abundance of TEP and CSP were similar. Even though the overall concentration and area of gel particles was not significantly different between regimes, CSP area was enriched in the open and coastal stations, while it was rather depleted in the upwelling stations in the SML. These results indicate that enrichments seem not only to depend on the concentration of gel particles, but also on the system that the SML is covering and the abiotic factors, e.g., wind speed, radiation. Another factor influencing the enrichment of gel particles in the SML might be the concentration of their precursors in the SML and the ULW and the abundance of microorganisms degrading the gel particles.

TEP abundance and area were almost depleted in the ULW at the open ocean stations while TEP were still abundant in the SML (Figure 4A). Previous studies who found difference in the SML enrichment of gel particles among oligotrophic and eutrophic habitats suggested that solid particles are less abundant over oligotrophic habitats resulting in less aggregation with TEP and thus less sinking of TEP aggregated with heavier particles (Jennings et al., 2017). Another factor influencing the high EF of TEP in the open ocean stations could be the formation of TEP *in situ* in the SML due to e.g., high wind speeds and thus high shear and likely formation of microwaves at the sea surface which can lead to an aggregation of TEP precursors as suggested by previous studies (Wurl et al., 2011a). The wind speeds recorded over the open ocean stations were among the highest during the cruise (Table 1).

As mentioned above, we observed high irradiance at the first two stations in the open ocean. At the same stations, exceptionally high EFs of TEP and TCHO were observed. Also the two highest bacterial enrichments were observed at these two stations which seems counterintuitive. TEP concentrations in the water can be influenced by UV radiation in two ways: either the UV light stimulates microorganisms to produce protective compounds (as indicated by the MAA absorption peaks at stations 1 and 2, Figure 5), which leads to an increased TEP formation, or it decreases TEP abundance by photodegradation (Wurl et al., 2011a). Laboratory studies have shown that UV B radiation can degrade TEP by photolysis, but also stimulates TEP release by microorganisms, possibly due to enhanced secretion of photo protective compounds or cell death (Orellana and Verdugo, 2003; Ortega-Retuerta et al., 2009). The higher enrichment of bacteria at stations with high UV radiation seems surprising, but this might be due to bacterial colonization of TEP which provide UV protection and a substrate for bacteria in the SML, a role which has been suggested previously (Mari and Kiørboe, 1996; Ramaiah et al., 2000; Passow, 2002). The effect of UV light on bacterial communities is not yet fully understood and while some studies did find effects, others found no effects of UV light on the microbial community (Bailey et al., 1983; Carlucci et al., 1985; Agogué et al., 2005b).

A number of studies have shown an enrichment of carbohydrates and proteinaceous material in the SML in a variety of habitats from arctic to tropical regions (Kuznetsova and Lee, 2001; Gao et al., 2012; Cunliffe et al., 2013; Engel and Galgani, 2016). Also gel particles, such as TEP, have shown an enrichment in the SML across different systems (Wurl et al., 2011a; Cunliffe et al., 2013). These enrichments of different components of the organic matter in the SML indicate that the SML is a distinct habitat with different properties compared to the ULW. However, to our knowledge, these parameters were usually measured and analyzed separately, while in this study we took all biogenic parameters and combined them to analyze the differences between the SML and the ULW (Figure 6). Our analysis indicates that the water in the SML is more similar between stations, rather than between the SML and the ULW of a single station.

This has important implications for sampling the surface ocean and inferences made from CTD samples on the ocean-atmosphere gas exchange. The gas exchange between the ocean and the atmosphere is influenced by the physicochemical composition of the SML (Upstill-Goddard et al., 2003; Frew, 2005; Salter et al., 2011). Considering that the Peruvian upwelling region is a net source of important greenhouse gases such as CO_2_, CH_4_, and N_2_O (Friederich et al., 2008; Naqvi et al., 2010; Arevalo-Martínez et al., 2015), it is even more important to understand the influence of the SML. The clear enrichment of DOM in the SML can potentially suppress the gas transfer velocity as has been shown in laboratory experiments (Goldman et al., 1988; Frew et al., 1990). The DOM concentration in the SML however will not be elucidated by solely taking samples from the CTD, instead, the SML has to be sampled directly.

### High organic matter enrichment in the SML of the open ocean

The overall concentrations of TOC and DOC are comparable with what has previously been found in the Peruvian upwelling region (Engel and Galgani, 2016). However, differences between the enrichment in the three regimes were observed. Strikingly, the highest TOC and DOC concentrations were found in the SML of the open stations.

One transport mechanism for DOM as well as gel particles from the ULW to the SML is through bubble scavenging (Aller et al., 2005; Wurl and Holmes, 2008). The highest wind speeds during SO243 were observed at the open stations, possibly resulting in a high bubble formation rate and thus large transport of DOC from the ULW to the SML.

In contrast to the DOC concentrations in the SML, the concentrations in the ULW were similar across the different regimes supporting the bubble scavenging hypothesis further if we assume that the transport from the ULW to the SML is small in comparison to the DOC formation rate in the ULW. If the ULW reservoirs are comparable between the different regimes, the transport in the open ocean to the SML has to be larger.

DOC concentrations in the surface ocean at ~5 m depth have been analyzed recently with regard to the influence of oligotrophic and eutrophic systems (Quinn et al., 2014). DOC concentrations across different systems were similar which lead to the conclusion that local system productivity and the recent history of primary productivity does not influence the DOC concentration in the surface (Quinn et al., 2014). The similar DOC concentrations in the ULW that we found across the different regimes are in accordance with this theory. For the DOC concentrations in the SML, however, it seems that different driving factors are important.

The same pattern was observed for T-/DCHO, T-/DAA, and FAA. All these OM components have the highest SML enrichments in the open ocean. A high enrichment of OM such as TOC and TEP over the open ocean has been also observed in previous studies (Wurl et al., 2011b; Jennings et al., 2017). Strikingly the degradation state of the OM calculated from the proportion of DOC that is made up of DAA, also follows the same trend. The OM in the SML is generally less degraded compared to the ULW. This difference is most pronounced in the open ocean, whereas the difference between SML and ULW decreases in the coastal stations and is not observed in the upwelling stations anymore.

The low degradation of OM could be a result of either higher phytoplankton activity producing more labile DOM, or decreased bacterial activity leading to a decrease in OM degradation and a higher retention time of labile DOM in the SML.

As mentioned above, TrL were enriched in the SML. This could be indicative of a higher phytoplankton activity of the diazotroph *Trichodesmium* in the SML. In any case, together with the high% DAA, this shows that not all the labile OM in the SML of the open stations is used by microorganisms even though the open ocean is possibly more OM limited than the coastal or the upwelling region. One explanation could be another limiting factor such as N or Fe influencing the microbial activity, which could result in an enrichment of diazotrophic phytoplankton.

It has been suggested that OM in the SML is removed by formation of sinking particulate OM (Wurl et al., 2011b). During SO243, the enrichment of TEP across the different regimes showed similar trends to that of organic matter which would support this hypothesis.

## Conclusions and outlook

Considering the fact that the open ocean makes up the majority of the marine environment, the higher enrichment of organic matter in the SML of the open ocean stations potentially has a significant influence on marine biogeochemical cycles. As mentioned above, numerous studies have shown the effect that surfactants in the SML have on the air-sea gas exchange (Upstill-Goddard et al., 2003; Frew, 2005; Salter et al., 2011). A SML enrichment of surfactants decreasing the gas transfer velocity between the ocean and the atmosphere over the open ocean would lead to an overestimation of the gas exchange when only looking at deeper waters.

Further investigations are needed to examine the differences in SML enrichment between different systems with an emphasis on open ocean vs. coastal sites to quantify the differences in gas transfer velocity and thus the impact on global biogeochemical cycles. Another factor not analyzed during our study is the atmospheric input of e.g., iron which can drive surface primary productivity (Young et al., 1991; Sarthou et al., 2003). Thus, further studies should also include the analysis of atmospheric input, such as dust, on the organic matter and microbial community composition of the SML.

## Author contributions

AB was responsible for the sampling and analysis of the HPLC data at 6 m used to characterize the different regimes. RR conducted the sampling and measurement of the absorption of the dissolved and particulate material in the SML and the underlying water. AE was involved in the analysis of the organic matter and microbial data. BZ was responsible for sampling and analyzing the organic matter and microbial community. All authors were involved in the writing and reviewing of the manuscript.

### Conflict of interest statement

The authors declare that the research was conducted in the absence of any commercial or financial relationships that could be construed as a potential conflict of interest. The reviewer ADS and handling Editor declared their shared affiliation.
